# How to write a Doctoral Thesis

**DOI:** 10.12669/pjms.322.10181

**Published:** 2016

**Authors:** HR Ahmad

**Affiliations:** Prof. HR Ahmad, Department of Biological and Biomedical Sciences, The Aga Khan University, Karachi, Pakistan. E-mail: hrahmad.alrazi@aku.edu; ***Note:*** * Ahmad HR. In: Medical Writing. Eds. SA Jawaid, MH Jafary & SJ Zuberi. PMJA, 1997 Ed II: 133-142.

PATIENT care and teaching are rather well established components of our medical career. However, with the passage of time a third component has started to influence our medical culture, namely research.^1^-^4^ How to accept this challenge is a question.^5^ Indeed, teaching and research form a dialectic unit, meaning that teaching without a research component is like a soup without salt. It is a well-established fact that the research activity of an institution is directly proportional to the number of qualified and committed PhD candidates. An inspiring infrastructure, laboratory facilities and libraries are pre-requisites for a research culture to grow.^6^-^8^ This forms the basis of a generation cycle for an institution, so that research activity and its culture continues to grow from one generation to the next. The main objective of doctoral work in biomedical sciences is to develop a galaxy of scientist physicians and surgeons possessing high degree of humility, selflessness and ethical superiority. Such a programme will add a scholastic dimension to the clinical faculty.

Education in how to write a doctoral thesis or dissertation should be a part of the postgraduate curriculum, parallel to the laboratory work and Journal Club activities during the PhD studies and/or residency levels.^9^,^10^ The overall structure of a doctoral thesis is internationally standardized. However, it varies in style and quality, depending upon how original the work is, and how much the author has understood the work. Therefore a thorough discussion with supervisor, colleagues and assistance from other authors through correspondence can be useful sources for consultation.

## Selection of a Topic

The choice of a topic for a doctoral thesis is a crucial step. It should be determined by scanning the literature whether the topic is original or similar work has already been done even a hundred years ago. It is the responsibility of both the supervisor and the PhD candidate to sort out this problem by continuous use of internet and a library.^11^ The work leading to the PhD degree can originate from research in following spheres:^12^

a) Basic
b) Methodology
c) Diagnostic
d) Therapeutic and Management
e) Epidemiology

The availability of internationally standardized methods, as well as research committed supervisors can enable physicians and surgeons to do PhD work in both basic and clinical health sciences. The importance of research in basic health sciences cannot be overemphasized. It is rather the base of the applied sciences. There are many instances where the elucidation of a mechanism involved in a process awaits the development of an adequate methodology.^13^ In such a scenario; a new method is like a new eye. Research activity in the field of (a) and (b) illuminates the research directions for (c) (d) and (e). It is worth noting that sometimes important basic questions can come from (e) and stimulate research activity in the domain of basic health sciences.^14^,^15^

## Types of Doctoral Thesis

**TYPE-I:** Book Form: a classical style. The blueprint of this form is shown in Table-I.

**Table-I:** Type-I: The Classical Book Form

INTRODUCTION:	Literature review. Identification of unresolved problem Formulation of aims and objectives.
METHODOLOGY:	Design. Outcome variable. Statistical analysis.
RESULTS:	Figures and tables with appropriate legends. Description, though not explanation of figures.
DISCUSSION:	Criticism of methodology and design Important observations. Interpretation and reasoning of results. Staging debate with the data of a literature table.
CONCLUSION:	Based on the premises of outcome. Claim of original research. Implications for future research directions.
REFERENCES:	Well analyzed.

**TYPE-II:** Cumulative Doctoral thesis: A modem but quite useful practice.

## INTRODUCTION

A book containing the pearls of a PhD work has standardized divisions and formats, where the number of pages should be weighted in terms of content rather than container. The book includes summary, introduction, materials and methods, results, discussion, conclusions, references and acknowledgements.

Two exercises are mandatory before starting a PhD programme:

- Literature survey using a regular library hours and internet surfing
- Familiarization with the hands-on-experience of methodology involved in the work
- The importance of a continuous literature survey using library, internet and direct correspondence with authors across the globe in the same field cannot be over-emphasized. The main goal of this exercise is to pinpoint the unresolved problem in the literature. An attempt to solve this problem now becomes the topic of the PhD thesis. All the relevant references should be collected, and carefully preserved in the form of a card system arranged alphabetically according to themes and authors. The introduction of the thesis should be styled like a review article with a critical analysis of the work of authors in the literature. The aims of the present PhD work can then also be addressed in the form of questions. The objectives would then deal with how to achieve the aims of the proposed study.

## MATERIALS / SUBJECTS AND METHODS

Now comes the most crucial and functional part of the doctoral work, the materials/subjects and methods section. This part can be considered as the motor of the PhD work. The reliability, sensitivity and specificity of the motor must be checked before embarking on a long journey. Controlling the controls is the best guide for a precise and authentic work. Usually materials and methods contain components such as a description of the species involved, their number, age, weight and anthropometric parameters, types of surgical procedures and anesthesia if applied, and a detailed description of methodology. Continuous or point measurements should be thoroughly described. However, a dynamic method should always be preferred to static one.

The experimental protocol should be designed after a small pilot study, which is especially advisable in research on human subjects. A detailed and well-thought experimental protocol forms the basis of conditions under which the results would be obtained. Any deviation from the experimental protocol will affect the outcome, and the interpretation of results. It may be noted that great discoveries are usually accidental and without a protocol, based merely on careful observation! However, for the sake of a publication, a protocol has to be designed after the discovery. After having described the different phases of the experimental protocol with the help of a schematic diagram e.g., showing variables, time period and interventions, the selection of a statistical method should be discussed. Negative results should not be disregarded because they represent the boundary conditions of positive results. Sometimes the negative results are the real results.

It is usual practice that most PhD candidates start writing the methodological components first. This is followed by writing the results. The pre-requisites for writing results are that all figures, tables, schematic diagrams of methods and a working model should be ready. They should be designed in such a way that the information content of each figure should, when projected as a frame be visually clear to audience viewing it from a distance of about fifty feet. It is often observed that the presenters themselves have difficulty in deciphering a frame of the Power-Point being projected in a conference.

## RESULTS

The results of a doctoral thesis should be treated like a bride. The flow of writing results becomes easier if all figures and tables are well prepared. This promotes the train of thoughts required to analyze the data in a quantitative fashion. The golden rule of writing results of a thesis is to describe what the figure shows. No explanation is required. One should avoid writing anything which is not there in a figure. Before writing one should observe each diagram for some time and make a list of observations in the form of key words. The more one has understood the information content of a figure; the better will be the fluency of writing. The interruption of the flow in writing most often indicates that an author has not understood the results. Discussion with colleagues or reference to the literature is the only remedy, and it functions sometimes like a caesarean procedure.

Statistical methods are good devices to test the degree of authenticity and precision of results if appropriately applied. The application of statistical technique in human studies poses difficulties because of large standard deviations. Outliers must be discussed, if they are excluded for the sake of statistical significance. Large standard deviations can be minimized by increasing the number of observations. If a regression analysis is not weighted, it gives faulty information. The correlation coefficient value can change from 0.7 to 0.4 if the regression analysis is weighted using Fisher’s test. The dissection of effect from artifact should be analysed in such a way that the signal to noise ratio of a parameter should be considered. A competent statistician should always be consulted in order to avoid the danger of distortion of results.

The legend of a figure should be well written. It contains a title, a brief description of variables and interventions, the main effect and a concluding remark conveying the original message. The writing of PhD work is further eased by a well maintained collection of data in the form of log book, original recordings, analyzed references with summaries and compiling the virgin data of the study on master plan sheet to understand the original signals before submitting to the procedures of statistics. The original data belong to the laboratory of an institution where it came into being and should be preserved for 5-7 years in the archive for the sake of brevity.

## DISCUSSION

This is the liveliest part of a thesis. Its main goal is to defend the work by staging a constructive debate with the literature. The golden rule of this written debate should be that a rigid explanation looks backward and a design looks forward. The object is to derive a model out of a jig-saw puzzle of information. It should be designed in such a way that the results of the present study and those of authors from the literature can be better discussed and interpreted. Agreement and disagreement can be better resolved if one considers under what experimental conditions the results were obtained by the various authors. It means that the boundary conditions for each result should be carefully analyzed and compared.

The discussion can be divided into the following parts:

- criticism of material/subjects and methods
- a list of important observations of the present study
- interpretation and comparison of results of other authors using a literature table
- design of a model
- claim of an original research work
- The criticism of the methodological procedure enables a candidate to demonstrate how precisely the research work has been carried out. The interpretation of results depends critically on the strict experimental protocol and methods. For example, an epidemiological work is a study of a population. However, if the population sampling is done regularly at a specific location; the question arises as to how a result derived from a localized place can be applied to the whole population.
- After having discussed at length the strong and weak points of material/subjects and methods, one should list in a telegraphic design the most important observations of the present study. This may form a good agenda to initiate interpretation, argument, reasoning and comparison with results of other authors. The outcome of this constructive debate should permit the design of a working model in the form of a block diagram. All statements should be very carefully referenced. The ratio of agreement and disagreement should indicate the ability of the author to reconcile conflicting data in an objective and quantitative way. Attempts should be made to design a solution out of the given quantum of information. It is also well known that most of the processes of human physiology can only be understood if their time course is known. The dynamic aspect of interpretation of results is therefore more powerful and superior to the static one.^16^ Therefore a continuous record of variables should be preferred and sought to reveal the secrets hidden in the kinetics.
- Finally, the discussion should conclude how far the study was successful in answering the questions being posed at the end of the introduction part. Usually a doctoral thesis raises more questions than it answers. In this way research does not come to a standstill and does become a life time engagement for a committed scientist. Also it is important to note that all scientific theses should be quantifiable and falsifiable, otherwise they lose the spirit and fragrance of a scientific research.
- The author’s claim of original work is finally decided by the critical review of his research work by the literature and the number of times being cited. It can be easily read by a high rate of a citation index of a publication and invitation. When a methodological research clicks, one becomes a star overnight.

## Type-II: CUMULATIVE DOCTORAL THESES

Another way of writing a doctoral work is a cumulative type of thesis.^11^ It consists of a few original publications in refereed journals of repute. It is supplemented by a concise summary about the research work. This type of thesis is usually practiced in Sweden, Germany and other countries. It has the advantage of being doubly refereed by the journals and the faculty of health sciences. Additionally, papers are published during a doctoral work. A declaration has to be given to the faculty of science about the sharing of research work in publications, provided there are co-authors. The weightage should be in favour of the PhD candidate, so that the thesis can ethically be better defended before the team of august research faculty.
